# Sex differences in left ventricular stroke work and cardiac power output per unit myocardium relate to blood pressure in apparently healthy adults

**DOI:** 10.1371/journal.pone.0280143

**Published:** 2023-01-06

**Authors:** Jing Lu, Lixue Yin

**Affiliations:** 1 School of Medicine, University of Electronic Science and Technology of China, Chengdu, P.R. China; 2 Department of Cardiovascular Ultrasound and Non-invasive Cardiology, Sichuan Academy of Medical Sciences & Sichuan Provincial People’s Hospital, Chengdu, P.R. China; 3 Ultrasound in Cardiac Electrophysiology and Biomechanics Key Laboratory of Sichuan Province, Chengdu, P.R. China; York University, CANADA

## Abstract

**Background:**

Left ventricular stroke work per unit myocardium (LVSWM) and cardiac power output per unit myocardium (CPOM) are important measures of myocardial workload. The sex differences in the myocardial workload and its correlation with blood pressure remain largely unclear.

**Objectives:**

The purpose of this study is to investigate the sex differences in LVSWM and CPOM, and to relate them to blood pressure in a cohort of apparently healthy adults.

**Methods:**

The LVSWM and CPOM were estimated in 596 age- and heart rate-matched apparently healthy adults (298 men) using transthoracic echocardiography combined with cuff-measured brachial blood pressure. The data were compared between sexes, and the sex differences in LVSWM and CPOM were related to blood pressure.

**Results:**

After adjustment for the blood pressure, the LVSWM and CPOM were higher in women than in men [75.0 (73.7–76.4) vs 64.9 (63.5–66.2) cJ/100g for LVSWM, and 912.4 (894.1–930.6) vs 780.2 (762.0–798.5) milliwatt/100g for CPOM, respectively; all *P*<0.001]. After adjustment for the LVSWM and CPOM, the mean systolic and diastolic blood pressure were 7.4 mm Hg and 5.2 mm Hg higher in men than in women, respectively (all *P*<0.001).

**Conclusions:**

For any given blood pressure, the workload per unit myocardium is higher in apparently healthy women than in their male counterparts. A sex-specific definition of normal blood pressure with a relatively lower threshold for women can minimize the sex differences in the myocardial workload, which might reduce the potentially comparatively higher risk of heart failure in women.

## Introduction

The left ventricular (LV) contraction generates hydraulic energy to maintain systemic circulation. The amount of hydraulic energy equals the LV mechanical external work, which can be quantified with LV stroke work (LVSW) and cardiac power output (CPO) [1, 2]. The sarcomeres, which can be viewed as independent units of biological motors, are essential determinants of the mechanical properties of the heart [3]. For a normal heart, the amount of LV external work is directly proportional to the integrated power of these biological motors. Therefore, the LVSW and CPO are parameters of overall LV function, and cannot represent the truly myocardial performance or workload due to the individual difference in LV mass (LVM). The LVSW per unit myocardium (LVSWM) and CPO per unit myocardium (CPOM) are important measures of myocardial workload [4], which ensure the comparability between people with different LVM. To achieve maximum cardiac efficiency, the left ventricle and systemic vasculature must work in unison with each other [5]. Heart failure is not always an inherent myocardial or valvular disease, but it might be a consequence of the myocardial afterload mismatch [5].

Sex differences wildly exist in heart failure in terms of prevalence, incidence, and progression [6–8]. For example: 1) despite the similar prevalence of hypertension in both sexes, the risk of heart failure is greater in women than in men; 2) the prevalence of heart failure with preserved ejection fraction is higher in women than in men; 3) women have a higher risk of developing *de novo* heart failure after myocardial infarction than men; 4) women with *de novo* heart failure have worse survival rate than men; 5) apical ballooning syndrome is much more common in women than in men; and 6) women have a greater risk for acute low output syndrome than men. Moreover, a recent study found that for the same level of blood pressure, the risk of heart failure is higher in women than in men [9].

Consideration of sex differences in research studies would make an important impact on the development and testing of preventive and therapeutic interventions [10]. However, in the field of cardiology, little attention has been paid to the sex differences in the LV myocardial workload and the potential effect of blood pressure on them. Thus, the purpose of this study is to investigate the sex differences in LVSWM and CPOM, and to relate them to blood pressure in a cohort of apparently healthy adults using transthoracic echocardiography (TTE) combined with cuff-measured brachial blood pressure.

## Methods

### Study population

This study was conducted according to the World Medical Association Declaration of Helsinki. The Institutional Ethics Committee of both the Sichuan Academy of Medical Sciences & Sichuan Provincial People’s Hospital and School of Medicine, University of Electronic Science and Technology of China approved the study and waived the requirement for informed consent. A cohort of 596 apparently healthy, and age- and heart rate-matched adults (298 men) was sampled from a database of 3930 apparently healthy Chinese Han adults. All data in the database were collected from October 2017 to September 2020, and all subjects in the database were fully anonymized. The apparently healthy adults in the database were people who: 1) ≥ 18 years of age; 2) with normal body mass index (BMI) or overweight (overweight defined as BMI between 25 to 30 kg/m^2^); 3) showed normal or high blood pressure [high blood pressure defined as cuff-measured systolic blood pressure (SBP) ≥ 120 mm Hg and/or diastolic blood pressure (DBP) ≥ 80 mm Hg) during the data collection, but without a history of hypertension; 4) without any kind of known disease, and without any kind of known physical or mental disorders; 5) without alcohol or drug addiction; 6) were not professional athletes or pregnant women; 7) were not on any medication; 8) with normal TTE results; 9) with normal ECG results, which were examined just before or after the TTE; and 10) with normal blood tests results in red blood cell, hemoglobin, triglyceride, serum total cholesterol, high-density lipoprotein, low-density lipoprotein, fasting blood glucose, total protein, albumin, globulin, sodium, potassium, blood urea nitrogen and creatinine (the blood samples of each subject were taken and analyzed on the same day that the TTE was performed).

### Echocardiography, blood pressure measurement, and calculations

Comprehensive 2-dimensional TTE was performed with a Philips iU22, iE33, or EPIQ ultrasound system (Philips Medical Systems, Bothell, WA, USA); and the images were analyzed, measured, and interpreted according to the recommendations [11, 12]. The 2-dimensional TTE was used in this study because it is a generally accepted and the most wildly used technique in clinical practice, which has been recommended as a reliable technique in the assessment of cardiac structure and function [11, 12]. The body surface area (BSA) was calculated using the Mosteller formula. The BMI was calculated as the weight in kilograms divided by the square of height in meters squared. The fat-free body mass (FFM) was calculated according to the validated sex-specific formulas [13]. The LVM was calculated with the cube formula [11]. The LVMI was calculated as LVM divided by BSA. The LV end-diastole volume (LVEDV), LV end-systole volume (LVESV), and LV ejection fraction (LVEF) were derived from the biplane method of disks (modified Simpson’s rule) [11]. The measurements of LVEDV and LVESV for each participant were meticulously made in the LV apical 4- and 2-chamber views at end-diastole and end-systole, defined as the largest and smallest visible areas in each view, respectively. The stroke volume (SV) was calculated as LVESV subtracted from LVEDV. The LVEDVI, LVESVI, and SVI were calculated as LVEDV, LVESV, and SV divided by BSA respectively. Cardiac output (CO) was calculated as SV multiplied by heart rate (HR); cardiac index (CI) was calculated as CO divided by BSA.

The brachial artery blood pressure was measured just before the echocardiography using an automated cuff digital blood pressure monitor (Omron HBP-1300, Kyoto, Japan). The key steps for the blood pressure measurement included: 1) Avoided caffeine, exercise, and smoking for at least 30 minutes before measurement. 2) Had the participants relax, sitting in a chair (feet on the floor, back supported) for at least 5 minutes before the measurement. 3) The participants had emptied their bladders. 4) Neither the subjects nor the observers were allowed to talk during the rest period or during the measurement. 5) Removed all clothing covering the location of cuff placement. 6) Positioned the middle of the cuff on the upper arm at the level of the right atrium (the midpoint of the sternum), and the cuff was wrapped to a tightness that allows two fingers to be inserted in between the cuff and the arm. 7) Recorded BP in both arms, and used the arm that gave the higher reading for subsequent readings. 8) Then 2 repeated measurements with an interval of 2 minutes were taken, and the average of the 2 readings was used. The mean arterial blood pressure (MAP) was calculated as the SBP plus double DBP, then divided by 3. The pulse pressure (PP) was calculated as the SBP minus DBP.

To include both the steady and pulsatile power, we estimated CPO with Eq (1) [14]:

CPO=0.87×AoSBP×CO (1)

According to the invasive measurement validated relations between cuff-measured blood pressure and aortic blood pressure and the reference values of sex differences in pulse pressure amplification [15, 16], the aortic systolic pressure (AoSBP) in apparently healthy adults can be estimated with acceptable accuracy (AoSBP in man equals cuff-measured brachial SBP minus 7.3 mm Hg for man, and AoSBP in women equals cuff-measured brachial SBP minus 2.3 mm Hg) [17]. In Eq (1), the AoSBP in N/m^2^ (1 mm Hg = 133.322387415 N/m^2^) and the CO in m^3^/min (1 L = 0.001 m^3^) were used. Then the CPO in N*m/min was converted into milliwatts (1 N*m/min = 16.666666666666668 milliwatts). The CPOM in milliwatt/100g was calculated as the CPO in milliwatt divided by LVM in g, then multiplied by 100. The LVSW was calculated as the CPO in N*m/min divided by the HR ratio in beat/min. Then the LVSW in N*m was converted into cJ (1 N*m = 100 cJ). The LVSWM in cJ/100g was calculated as the LVSW in cJ divided by LVM in g, then multiplied by 100. The LVSW consists of the pressure work to maintain the blood pressure and the flow work to pump the SV [18, 19]. Thus, we calculated the MAP per unit myocardium (MAPM) and the SV per unit myocardium (SVM) to roughly quantify the LV myocardial pressure workload and flow workload, respectively. The MAPM in mm Hg/100g was calculated as MAP in mm Hg divided by LVM in g, then multiplied by 100. The SVM in mL/100g was calculated as SV in mL divided by LVM in g, then multiplied by 100.

Although the definitions of LV myocardial workload and LV myocardial afterload are completely different, the intrinsic connection between them should be noted. To quantify the LV myocardial afterload, we estimated the peak value of systolic myocardial fiber stress (Ó_ƒ_—peak) with Eq (2) [20],

o´f‐peak=P13ln(1+VWVC) (2)

where the V_W_ was the volume of the LV wall in cm^3^, and V_C_ was the volume of the LV chamber in cm^3^. The V_W_ was calculated as LVM divided by myocardial density (1.04 g/cm^3^). The change of V_C_ from end-diastole to the Ó_ƒ_—peak occurring is minimal [21]. And the Ó_ƒ_—peak usually occurs within 100 ms after the onset of systole and always follows the onset of LV dimensional reduction [21].Thus, the V_C_ in Eq (2) was substituted with LVEDV. The P in Eq (2) was the LV systolic pressure in kdynes/cm^2^ (1 mmHg = 1.33322368 kdynes/cm^2^) at the time point of Ó_ƒ_—peak occurring. The LV pressure at the time point of Ó_ƒ_—peak occurring is nearly 82% of the peak value of LV systolic pressure [22]. And the AoSBP is equivalent to the peak value of LV systolic pressure in the normal heart. Thus the P in Eq (2) for calculating the Ó_ƒ_—peak was estimated as 0.82 × AoSBP.

### Statistical analysis

Analyses were performed using SPSS Version 22 (IBM Corp., Armonk, NY). To reduce selection bias and improve internal validity, case-control matching was performed to sample the subjects from the database based on age in year and HR in beat/min with tolerance levels of 2 and 2, respectively (the categorical variable “sex” was chosen as the “Group Indicator”). Categorical variables were expressed as cases (percentage), and compared using Chi-square tests. Continuous variables with normal distribution were summarized as mean ± standard deviation, and those without normal distribution were summarized as median (interquartile range). Testing for normality was performed with the Kolmogorov-Smirnov test. Independent-samples *t*-tests were used to compare the mean of two independent samples with normal distribution, and nonparametric tests for two independent samples were used to compare the mean of non-normally distributed data. The general linear model was used to adjust the effects of the blood pressure or the LVM on the sex differences in the LVSWM and CPOM, and to adjust the effects of the LVSWM and CPOM on the sex differences in the blood pressure. The estimated marginal means (95% CIs) were compared between sexes after the adjustments, and the mean differences were computed. To assess the correlation between LV mechanical external work and LV myocardial afterload; linear correlation and regression analyses were performed between LVSWM and Ó_ƒ_—peak, and between CPOM and Ó_ƒ_—peak. Log transformation was performed when needed to satisfy the statistical assumption. A *P* value less than 0.05 was considered statistically significant.

## Results

### Basic characteristics of the study population

A total of 596 age- and HR-matched apparently healthy adults (298 men) were included, and the age ranged from 21 years to 65 years old. Among the 596 adults, overweight was found in 77 subjects (42 men), and high blood pressure was found in 154 subjects (78 men). The basic characteristics of the study population stratified by sex are presented in Table 1. No statistical difference was found in the age, HR, PP, percentage of overweight cases, and percentage of high blood pressure cases between sexes (all *P>*0.05). The height, weight, BSA, BMI, and FFM were larger in men than in women (all *P*<0.001). The SBP, DBP, and MAP were higher in men than in women (all *P*<0.001).

**Table 1:** Sex differences in basic characteristics, blood pressure, LV volume and mass, and LV external work in 596 apparently healthy adults.

	Men (n = 298)	Women (n = 298)	*Z*, *t*, or *χ*^*2*^	*P* value
**Basic characteristics**				
Age, yrs	45.0 (38.0–51.0)	45.0 (39.0–51.0)	0.579	0.562
Height, m	1.69 (1.65–1.72)	1.58 (1.54–1.62)	17.735	<0.001
Weight, kg	65.0 (61.0–70.0)	54.0 (51.0–59.0)	15.250	<0.001
BSA, m^2^	1.83 (1.76–1.90)	1.63 (1.57–1.69)	17.231	<0.001
Body mass index, kg/m^2^	23.1 (21.8–24.4)	22.0 (20.6–23.8)	4.895	<0.001
Overweight, n (%)	42 (14.1)	35 (11.7)	0.731^a^	0.393
Fat-free mass, kg	51.5 ± 3.8	39.3 ± 2.9	40.422^b^	<0.001
Heart rate, bpm	73.0 (70.0–75.0)	74.0 (70.0–75.0)	0.648	0.517
SBP, mm Hg	112.0 (104.0–119.0)	108.0 (99.0–118.3)	3.440	<0.001
DBP, mm Hg	69.0 (64.8–76.0)	66.0 (60.0–73.0)	4.676	<0.001
MAP, mm Hg	83.3 (78.3–90.0)	79.8 (74.0–88.7)	4.466	<0.001
PP, mm Hg	41.0 (36.0–47.0)	40.0 (36.0–48.0)	0.653	0.514
High blood pressure, n (%)	78 (26.2)	76 (25.5)	0.035^a^	0.852
**Echocardiographic measurements**				
AAO, mm	29.5 (28.0–31.0)	27.0 (25.0–29.0)	9.357	<0.001
LAD, mm	32.0 (30.0–33.0)	30.0 (29.0–32.0)	5.913	<0.001
IVS, mm	9.0 (8.0–10.0)	8.0 (7.0–9.0)	8.935	<0.001
LVPW, mm	9.0 (8.0–9.0)	8.0 (7.0–9.0)	8.621	<0.001
LVEDD, mm	45.0 (42.8–47.0)	43.0 (41.0–45.0)	7.433	<0.001
E, cm/s	78.5 (69.0–89.3)	86.5 (78.0–97.0)	6.299	<0.001
A, cm/s	60.0 (53.0–69.0)	64.0 (55.0–79.0)	3.284	0.001
e, cm/s	13.0 (11.0–15.0)	14.0 (12.0–16.0)	2.895	0.004
a, cm/s	9.0 (8.0–11.0)	9.0 (8.0–11.0)	1.079	0.281
E/a	6.1 (5.1–7.1)	6.2 (5.4–7.5)	2.040	0.041
LVEDV, mL	100.0 (89.4–109.9)	90.6 (81.7–100.0)	7.433	<0.001
LVEDVI, mL/m^2^	55.2 (48.9–60.3)	54.8 (50.0–61.1)	0.599	0.549
LVESV, mL	31.4 (27.6–35.7)	28.1 (25.3–32.1)	6.322	<0.001
LVESVI, mL/m^2^	17.1 (15.0–19.6)	17.3 (15.4–19.5)	0.564	0.573
LV ejection fraction, %	69.1 (66.3–70.2)	69.2 (67.1–70.5)	0.325	0.745
SV, mL	68.6 (60.3–75.9)	61.6 (55.0–68.0)	7.602	<0.001
CO, L/min	4.88 (4.34–5.45)	4.45 (4.03–4.95)	6.717	<0.001
SVI, mL/m^2^	37.6 ± 5.5	37.8 ± 5.2	0.532^b^	0.595
CI, L/min/m^2^	2.69 (2.42–2.96)	2.70 (2.46–3.03)	1.079	0.035
SV/FFM, mL/kg	1.34 ± 0.20	1.58 ± 0.22	14.033^b^	<0.001
CO/FFM, mL/min/kg	96.8 ± 15.2	115.0 ± 17.9	13.339^b^	<0.001
LVM, g	128.0 (113.6–148.1)	105.3 (88.5–122.3)	10.925	<0.001
LVMI, g/m^2^	71.4 ± 14.0	64.8 ± 12.9	6.011^b^	<0.001
LVM/FFM, g/kg	2.50 (2.19–2.89)	2.71 (2.24–3.09)	3.316	<0.001
**LV external work and myocardial afterload**				
LVSW, cJ	81.9 (71.4–93.9)	76.2 (65.8–85.1)	5.121	<0.001
CPO, milliwatt	984.4 (848.4–1150.2)	928.1 (793.5–1045.5)	4.389	<0.001
LVSWM, cJ/100g	63.4 (56.4–73.3)	71.7 (63.4–82.9)	7.226	<0.001
CPOM, milliwatt/100g	780.4 (672.2–898.1)	876.3 (746.8–1019.0)	6.662	<0.001
MAPM, mm Hg/100g	64.0 (56.6–77.0)	76.7 (67.1–91.5)	8.478	<0.001
SVM, mL/100g	52.1 (47.7–57.9)	59.0 (52.0–67.3)	7.621	<0.001
Ó_ƒ_—peak, kdynes/cm^2^	425.1 (389.9–472.0)	462.5 (419.5–520.6)	6.634	<0.001

Note: Data with normal distribution are expressed as mean ± standard deviation and those without as median (interquartile range). Independent-samples *t*-tests are used to compare the mean of two independent samples with normal distribution, and a *t*-value is presented. Nonparametric tests for two independent samples are used to compare the mean of non-normally distributed data, and a *Z* value is presented.

In the “*Z*, *t*, or *χ*^*2*^” column, ^a^ indicates a *χ* ^*2*^ value, b indicates a *t* value, and others are *Z* values.

LV, left ventricular; BSA, body surface area; SBP, brachial arterial systolic blood pressure; DBP, brachial arterial diastolic blood pressure; MAP, mean arterial pressure; PP, pulse pressure; LVEDV, LV end-diastolic volume; LVESV, LV end-systolic volume; SV, stroke volume; CO, cardiac output; CI, cardiac index; LVM, LV mass; LVSW, LV stroke work; CPO, cardiac power output; LVSWM, LVSW per unit myocardium; CPOM, CPO per unit myocardium; MAPM, MAP per unit myocardium; SVM, SV per unit myocardium. The LVEDVI, LVESVI, SVI, and LVMI are these parameters indexed to BSA respectively.

### Sex differences in the echocardiographic measurements and related variables

The sex differences in the echocardiographic measurements and related variables are presented in Table 1. The diameter of ascending aorta, left atrium and left ventricle were larger in men than in women; and the thickness of the interventricular septum and LV posterior wall were thicker in men than in women (all *P*<0.001). The LVEDV, LVESV, SV, CO, LVM, and LVMI were larger in men than in women (all *P*<0.001). No statistical difference was found in the LVEDVI, LVESVI, SVI, CI, and LVEF between sexes (Table 1). The SV/FFM, CO/FFM, and LVM/FFM were smaller in men than in women (all *P*<0.001).

### Sex differences in LV external work and myocardial workload

**Fig 1:**
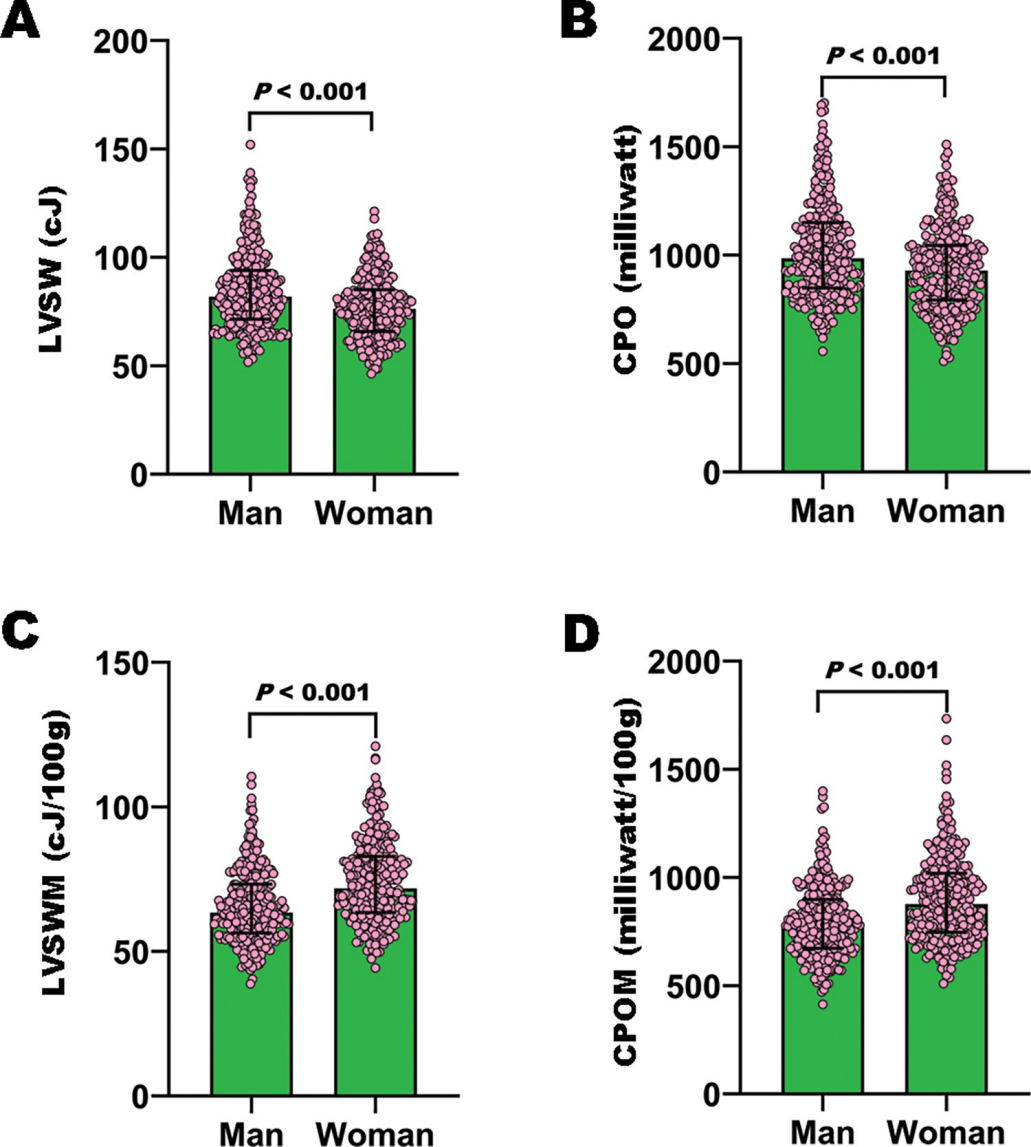
Sex difference in LV external work in 596 apparently healthy adults (298 men). The LVSW and CPO, which represent the overall LV function, are higher in men than in women (Fig A and B). The LVSWM and CPOM, which represent the workload per unit LVM, are higher in women than in men (Fig C and D). Nonstandard abbreviations and acronyms as in Table 1.

The LVSW and CPO were higher in men than in women (all *P*<0.001); but the LVSWM, CPOM, MAPM, and SVM were higher in women than in men (all *P*<0.001) (Table 1 and Fig 1A–1D). The LVSW and CPOM were higher in women than in men after the adjustment for the cuff-measured SBP and DBP (all *P*<0.001); but the sex differences in these parameters disappeared after the adjustment for the LVM (all *P*>0.05) (Table 2). After the adjustment for the LVSWM and CPOM, the mean SBP and DBP were 7.4 mm Hg and 5.2 mm Hg higher in men than in women, respectively (all *P*<0.001) (Table 3).

**Table 2:** Sex differences in LVSWM and CPOM in 596 apparently healthy adults after adjustment for the blood pressure or the LVM.

	Men (n = 298)	Women (n = 298)	Mean difference	*P* value
**Adjusted to SBP and DBP**				
LVSWM, cJ/100g	64.9 (63.5–66.2)	75.0 (73.7–76.4)	10.2 (8.2–12.1)	<0.001
CPOM, milliwatt/100g	780.2 (762.0–798.5)	912.4 (894.1–930.6)	132.2 (106.1–158.2)	<0.001
**Adjusted to LVM**				
LVSWM, cJ/100g	69.3 (67.9–70.7)	70.6 (69.2–72.0)	1.3 (-0.8–3.4)	0.215
CPOM, milliwatt/100g	838.0 (819.0–857.1)	854.6 (835.5–873.6)	16.6 (-11.8–44.9)	0.252

Data are estimated marginal means (95% CIs). Nonstandard abbreviations and acronyms as in Table 1.

**Table 3:** Sex differences in blood pressure in 596 apparently healthy adults after adjustment for the LVSWM and CPOM.

	Men (n = 298)	Women (n = 298)	Mean difference	*P* value
SBP, mm Hg	114.8 (113.5–116.1)	107.3 (106.0–108.6)	7.4 (5.5–9.3)	<0.001
DBP, mm Hg	71.6 (70.6–72.5)	66.4 (65.4–67.3)	5.2 (3.8–6.5)	<0.001
MAP, mm Hg	86.0 (85.0–86.9)	80.0 (79.1–81.0)	5.9 (4.5–7.3)	<0.001
PP, mm Hg	43.2 (42.2–44.2)	41.0 (40.0–42.0)	2.2 (0.8–3.7)	0.002

Data are estimated marginal means (95% CIs). Nonstandard abbreviations and acronyms as in Table 1.

### Correlation between LV myocardial workload and afterload

**Fig 2:**
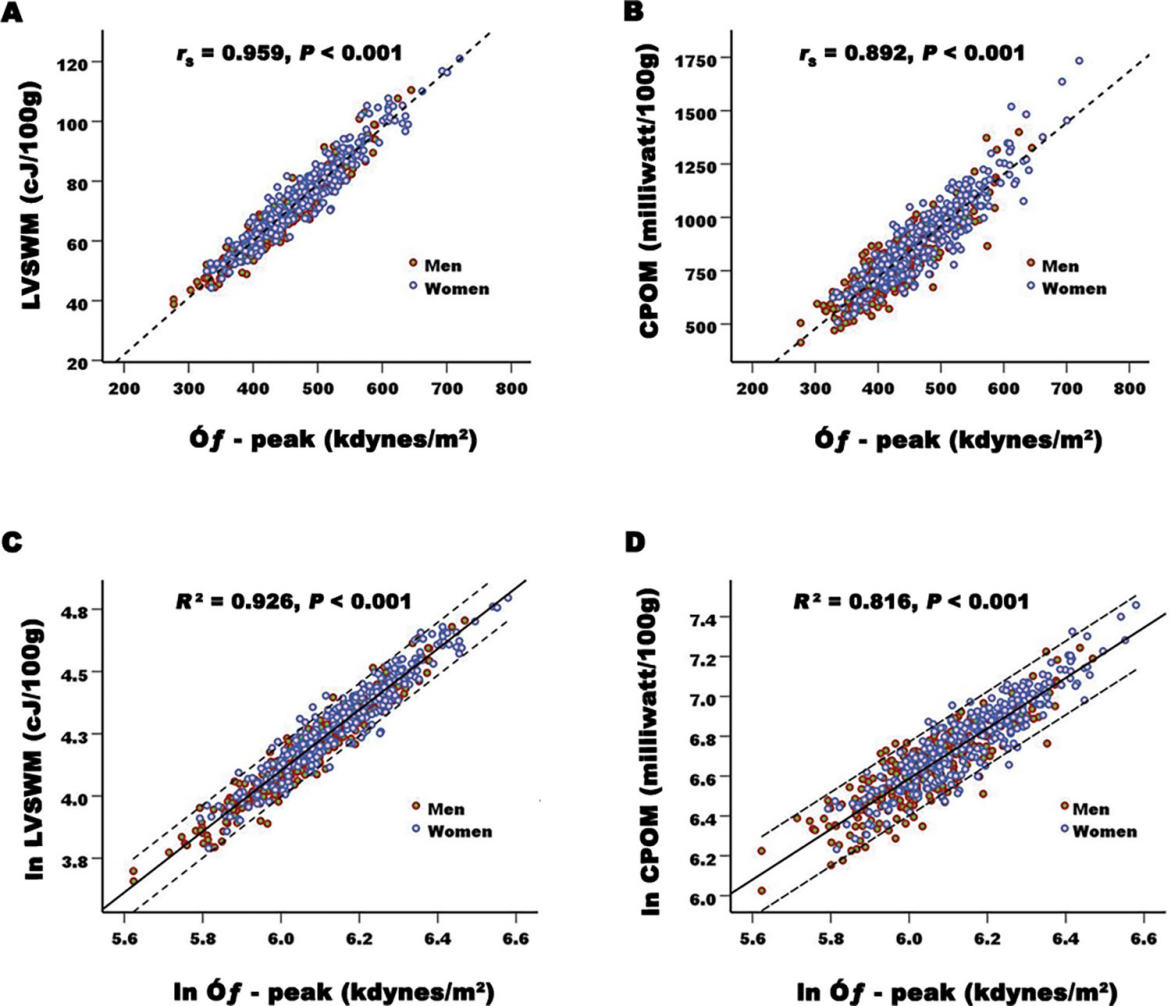
Linear correlation and regression between the LVSWM and Ó_ƒ_—peak, and between CPOM and Ó_ƒ_—peak in 596 apparently healthy adults (298 men). Very strong correlation exists between the LVSWM and the Ó_ƒ_—peak (A), and a strong correlation exists between the CPOM and the Ó_ƒ_—peak (B). The natural logarithm of LVSWM, CPOM, and the Ó_ƒ_—peak are normally distributed data. C and D show that both the LVSWM and the CPOM increase with Ó_ƒ_—peak (ln LVSWM = -3.233 + 1.223 × ln Ó_ƒ_—peak; and -1.007 + 1.266 × ln Ó_ƒ_—peak). These figures indicate that the higher the LV myocardial afterload is, the more work has to be done by per unit myocardium. Abbreviations as Table 1.

The correlation between LV myocardial workload and afterload is showed in Fig 2. In the 498 apparent healthy adults, a very strong positive correlation was found between the LVSWM and the Ó_ƒ_—peak (*r*_*s*_ = 0.959, *P*<0.001) (Fig 2A), and a strong positive correlation was found between the CPOM and the Ó_ƒ_—peak (*r*_*s*_ = 0.892, *P*<0.001) (Fig 2B). The natural logarithm of the LVSWM, CPOM, and Ó_ƒ_—peak were normally distributed data, which were used in the simple linear regression analyses. Both the LVSWM and the CPOM increased with the Ó_ƒ_—peak (Fig 2C and 2D). The regression equations were: ln LVSWM = -3.233 + 1.223 × ln Ó_ƒ_—peak, and ln CPOM = -1.007 + 1.266 × ln Ó_ƒ_—peak (*R*^2^ = 0.926 and 0.816 respectively, all *P*<0.001).

## Discussion

The sex differences in LV myocardial workload in a cohort of middle-aged apparently healthy adults are investigated using TTE combined with cuff-measured brachial blood pressure. The primary findings include: 1) The LV myocardial workload is higher in women than in men; 2) For any given blood pressure, the LV myocardial workload is higher in women than in men; 3) For the same amount of LV myocardial workload, the SBP and DBP are lower in women than in men.

### Sex differences in LV volumes and mass, and the effect of indexation

It is a typical feature that sexual dimorphism exists in body size and composition due to the differences in genes, hormones, and evolution. The LV volume and mass strongly relate to body size, indicating the need for appropriate normalization. The FFM includes organ cell mass and nonfatty tissues (tendons, ligaments, and bone) [23]. Up to 99% of body metabolism takes place in the FFM [23]. Given the intimate relation between CO and the level of metabolism, FFM-indexed-CO might be more sensitive than those BSA-indexed-CO for the true distinction between sexes. One strong heart study showed that the LVEDV, LVESV, SV, and CO are larger in men than in women; no statistical difference exists in the LVEDVI, LVESVI, SVI, and CI between sexes; and the SV/FFM and CO/FFM are considerably smaller in men than in women [23]. The researchers supposed that the higher SV/FFM and CO/FFM in women than in men might have been a compensation for lower hemoglobin concentration in women [23]. Another strong heart study found that the LVM is more strongly related to FFM than to adipose mass, and the LVM/FFM appears to increase the sensitivity for the detection of LV hypertrophy [24]. Their results showed that the FFM, LVM, and LVMI are much higher in men, but the LVM/FFM is small in men than in women [24]. Our results showed that the sex differences in the LVEDV, LVESV, SV, CO, and LVM in Chinese Han adults are entirely consistent with those in American Indians obtained in the strong heart studies, regardless of the indexation with BSA or with FFM involved.

### Sex differences in LV myocardial workload

It is almost impractical to quantify the workload of a single sarcomere or myocardial fiber in vivo. The metrics of LVSWM and CPOM provide the measurement of myocardial workload and ensure the comparability of myocardial performance between people with different LVM. This is the first study to our knowledge to investigate the sex differences in LV myocardial workload. And we found that the LV myocardial workload (both LVSWM and CPOM) is significantly higher in women than in men. The myocardial workload is a fundamental determinant of myocardial energy consumption [18, 19]. A previous study reported that both baseline and hyperaemic myocardial energy consumption in women are typically higher as compared to men [25], which is a piece of indirect evidence that the LV myocardial workload is higher in women than in men.

### Possible reasons for the sex differences in LV myocardial workload

Relatively higher LV afterload in women might be the essential reason for the sex differences in LV myocardial workload (LVSWM and CPOM). In an intact cardiovascular system, the LV myocardial workload is determined by myocardial shortening against myocardial tension and LV ejection against arterial load. Thus, theoretically, the LV myocardial workload increases with the afterload. The myocardial tension, namely LV myocardial afterload, can be quantified with LV wall stress or myocardial fiber stress. Chirinos et al. found that although the blood pressure is slightly higher in men, women demonstrate greater LV myocardial afterload than men in a large population of apparently healthy adults from two Belgian communities [21]. In another study, Phua et al. found that women have paradoxical higher LV wall stress at end-systole despite lower LVM than men in a group of Singapore adults without apparently cardiovascular conditions [26]. We found that the LV afterload (Ó_ƒ_—peak) is higher in women than men. Furthermore, we found that both LVSWM and CPOM are closely and positively correlated with the LV afterload. All of this evidence indicates that the relatively higher LV myocardial workload in women is closely related to their comparatively higher myocardial afterload.

The LVSWM and CPOM are the function of SV, CO, LVM, and blood pressure. Therefore, changes in any of these variables will change the amount of LV myocardial workload. As shown in the related equations, the LVSWM or CPOM decreases with decreasing SV, CO, and/or SBP. On the contrary, the LVSWM or CPOM increases with decreasing LVM. We found that women have lower SV, CO, and SBP than men, which is consistent with the previous study [23]. Thus, for the same LVM, the LV myocardial workload will be lower in women than in men. However, we found that both the LVSWM and CPOM are higher in women than in men. Therefore, the only possible explanation is women have a much lower LVM than men. This deduction is reasonable because women do have a significantly smaller LVM than men [13, 24, 27, 28]. Furthermore, we found that after the adjustment for the LVM, the sex differences in the LVSWM and CPOM disappeared. All the evidence demonstrates that the sex difference in LVM is an important determinant of the sex differences in the LV myocardial workload.

### The intrinsic connection between myocardial workload, blood pressure, and afterload

Blood pressure plays a pivotal role in modulating both myocardial workload and afterload. As you can see from the related equations, the blood pressure (AoSBP) is a common factor of both myocardial workload (LVSWM and CPOM) and afterload (Ó_ƒ_—peak). Thus, an increase in blood pressure indicates a proportional increase in both myocardial workload and afterload. The underlying mechanism of the intrinsic connection could be explained by the 3rd Law of Newton (Whenever an object exerts an action force on another, the latter simultaneously exerts a reaction force of equal magnitude and opposite direction on the first.). Pressure is the physical amount of force that is dispersed in one region, while force is the overall effect of one element on another. In an intact cardiovascular system, the blood pressure or LV systolic pressure in mm Hg dispersed on the LV endocardium during the systole can be converted into force in N/m^2^ (1 mm Hg = 133.322387415 N/m^2^). The LV contraction generates an action force to drive blood into the aorta and develop blood pressure in the relatively closed circulation system (the LV external work). Accordingly, the blood pressure exerts a reaction force of equal magnitude and opposite direction against the LV wall simultaneously (myocardial afterload). At any time point during the LV systole, there is always a dynamical action-reaction pair at the LV endocardium. These dynamical action-reaction pairs are the essential prerequisite for the formation of the LV myocardial workload (LVSWM and CPOM) and afterload (myocardial fiber stress). Therefore, both myocardial workload and afterload will decrease with the decreasing blood pressure simultaneously. The most extreme example is cardiac arrest, where the blood pressure, LV myocardial workload and myocardial afterload will disappear altogether.

### The potential effect of current non-sex-specific definitions of hypertension on the sex difference in LV myocardial workload and its clinical relevance

The non-sex-specific definitions of normal blood pressure and the non-sex-specific classification of arterial hypertension have been recommended and widely used [29]. We found that for any given blood pressure, more work needs to be done by per unit myocardium in apparently healthy women than in their male counterparts. The evidence indicates that women have a less efficient ventricular-arterial coupling or mismatched afterload for the same level of blood pressure. Furthermore, the non-sex-specific definitions of normal blood pressure neglect the fact that normal blood pressure in mid-aged healthy adults is typically higher in men than in women [30, 31]. Therefore, the non-sex-specific definitions of normal blood pressure factitiously impose a relatively higher myocardial workload and afterload in women. Considering that an excessive myocardial workload or a mismatched afterload is an essential reason for the risk of heart failure [5], the non-sex-specific definitions of hypertension might have contributed to the relatively higher risk of heart failure in women. In a seminal study, the researchers found 4081 subjects out of 27 542 participants (54% women) without baseline cardiovascular disease developed heart failure over 28±12 years follow-up [9]. They divided the blood pressure into 8 categories and found that the risk of heart failure is higher in women than in men for any level of blood pressure [9]. It appears from the evidence that the non-sex-specific classification of arterial hypertension is inappropriate for the management of high blood pressure in adults.

Arterial blood pressure is the most important and clinically controllable factor in modulating LV myocardial workload and afterload. Although LV myocardial workload is the function of LVM, SV, and CO, it might be unreasonable to factitiously change the intrinsic sexual dimorphism in LVM, SV, and CO under clinical conditions. Moreover, previous studies have confirmed that for any given LV mechanical external work, the myocardial oxygen consumption is much greater by increasing arterial blood pressure than by increasing CO [18, 19]. And myocardial tension (afterload) has been confirmed as the most important determinant of myocardial oxygen consumption [18, 19]. Finally, the normal blood pressure in mid-aged healthy adults is typically lower in women than in men [30, 31]. This evidence suggests that it is more practicable to use a sex-specific definition of normal blood pressure and the sex-specific classification of arterial hypertension, rather than attempting to change the intrinsic sexual dimorphism in LVM, SV, and/or CO, to reduce the sex differences in LV myocardial workload and afterload in clinical settings.

We found that after the adjustment for the LV workload (LVSWM and CPOM), the mean systolic and diastolic blood pressure were 7.4 mm Hg and 5.2 mm Hg lower in women than in men, respectively. Our results indicate that a relatively lower threshold of normal blood pressure for women can minimize the sex differences in LV myocardial workload and afterload, and might therefore reduce the comparatively higher risk of heart failure in women. Thus, we suppose that it is practical to establish a new sex-specific definition of normal blood pressure or ideal treatment target of hypertension for the management of high blood pressure in adults.

### Limitations

Firstly, the 3-dimensional TTE is free of geometric assumptions, and therefore proper usage of this technique might achieve more accurate data. Because the bias of the 2-dimensional TTE measurements here is similar for both sexes, thus the influence on our results is negligible. Secondly, although the accuracy of the estimated aortic blood pressure has been validated with invasive intra-arterial measurements in large groups of adults [15, 16], the estimated AoSBP might still slightly differ from the true values due to individual differences. Thirdly, our results are based on a group of middle-aged adults and may not fully justify alterations in blood pressure normative values for all women. Fourthly, the correlation between LV myocardial workload and afterload is expected; thus, caution is warranted given the co-linearity between these variables. Fifthly, invasive assessment of the intracardiac LV pressure-volume relationship is the gold standard to determine LVSW, and thus any other noninvasive method has its limitations. Lim suggested that the equation of CPO calculation that includes right atrial pressure (RAP) [CPO = (mean arterial pressure−RAP) × CO/451] is historically accurate, prognostically relevant, and practically applicable [32, 33]. Chemla et al. pointed out that although the use of the Guytonian approach to further understand cardiac hemodynamics is laudable, some limitations cannot be neglected [34]: 1) The Guytonian framework fails to express unilateral heart failure and resultant volume redistribution between the pulmonary circulation and the systemic circulation. 2) The formula proposed by Lim merely reflects the dissipated mean energy per unit time across the systemic circulation, which neglects the fact the heart is a pulsatile pump, not a steady pump. Furthermore, the omission of RAP has a negligible effect on CPO calculation under normal circumstances [32]. Thus, to take the pulsatile power into account, we used the formula proposed by Chemla et al. to estimate CPO for this cohort of apparently healthy adults. Lastly, although we supposed that the relatively higher myocardial workload (LVSWM and CPOM) in women is related to the less efficient ventricular-arterial coupling, we were not been able to validate it in this study by quantifying the ratio of arterial elastance to ventricular elastance.

### Perspectives

Our results only lay the cardiac mechanical groundwork for the proposition of a new sex-specific definition of normal blood pressure or ideal treatment target of hypertension for adults. Convincingly preclinical and clinical evidence is still needed to establish appropriately sex-specific thresholds for normal blood to reduce the sex differences in risk of adverse clinical outcomes. Our data is hypothesis-generating, which indicates that studies that use the incidence of chronic heart failure and the metrics of LV myocardial workload might be able to better answer the risk relationship.

The LVSW or CPO is the hydraulic energy associated with LV contraction and delivered to the systemic circulation per heartbeat or per minute, respectively. The LVSWM or CPOM represents the amount of work that has to be done by per unit myocardium (myocardial workload) per heartbeat or per unit time, respectively. The LVSW and CPO are parameters of overall LV function, but the LVSWM and CPOM represent the performance of unit myocardium or myocardial workload. Previous studies on the LVSW index, CPO index, or peak CPOM quantified with stress echocardiography has displayed important prognostic values of these parameters in cardiovascular disease [35–42]. Thus, it is hoped that this work will stimulate further research on these important measures of LV myocardial performance to improve the accuracy of cardiac function assessment and therefore to optimize clinical decisions in cardiovascular disease.

## Conclusions

For any given blood pressure, more work needs to be done by per unit myocardium in healthy women than in their male counterparts due to the intrinsic sex difference in LVM. Thus, the use of a non-sex-specific definition of normal blood pressure for both sexes factitiously mismatches a smaller LVM with a relatively higher myocardial workload in women. A sex-specific definition of normal blood pressure with a relatively lower threshold for women can minimize the sex differences in the myocardial workload, which might reduce the potentially comparatively higher risk of heart failure in women. Noninvasive assessment of LVSWM and COPM ensures the comparability of myocardial workload between people with different LVM under clinical conditions and provides a new insight into the myocardial performance.
